# ΔNp63α promotes cigarette smoke-induced renal cancer stem cell activity via the Sonic Hedgehog pathway

**DOI:** 10.1590/1678-4685-GMB-2023-0347

**Published:** 2024-07-01

**Authors:** Yuxiang Zhao, Nannan Ma, Wanngyu Wu, Ying Wu, Wenbo Zhang, Weiwei Qian, Xin Sun, Tao Zhang

**Affiliations:** Anhui Medical University, Second Affiliated Hospital, Department of Urology, Hefei, China; Anqing 116 Hospital, Department of Urology, Anqing, China

**Keywords:** Cigarette smoke, renal cancer stem cells, ΔNp63α, Sonic Hedgehog signaling pathway.

## Abstract

Cigarette smoke (CS) has been generally recognized as a chief carcinogenic factor in renal cell carcinoma (RCC). The stimulative effect of CS on renal cancer stem cells (RCSCs) has been described previously. The Sonic Hedgehog (SHH) pathway plays an essential role in self-renewal, cell growth, drug resistance, metastasis, and recurrence of cancer stem cells (CSCs). Renal cancer-related gene ΔNp63α is highly expressed in renal epithelial tissues and contributes to the RCSCs characteristics of tumors. The aim of this study was to elucidate the role of ΔNp63α and the SHH pathway on the activity of RCSCs induced by CS through a series of *in vivo* and *in vitro* studies. It was shown that in renal cancer tissues, ΔNp63α and RCSCs markers in smokers are expressed higher than that in non-smokers. RCSCs were effectively enriched by tumor sphere formation assay. Besides, CS increased the expression of RCSCs markers and the capability of sphere-forming *in vitro* and *in vivo*. Moreover, the SHH pathway was activated, and the specialized inhibitor alleviated the promotion of CS on RCSCs. ΔNp63α activated the SHH pathway and promoted CS-induced enhancement of RCSCs activity. These findings indicate that ΔNp63α positively regulates the activity of CS-induced RCSCs via the SHH pathway.

## Introduction

Renal cell carcinoma (RCC) is a common urinary malignancy. In 2020, there were about 77410 newly diagnosed cases and 46345 deaths in China and the United States (Xia et al., 2022). About 20%-30% of RCC has metastasis at the first time of diagnosis, and they are mostly resistant to radiotherapy and chemotherapy, leading to abysmal prognosis (Rouprêt et al., 2023). Cancer stem cells (CSCs) are cells in tumors that can self-renew and generate heterogeneous tumor cells, which play a crucial role in the formation and advancement of tumors (Wu et al., 2023). Therefore, exploring the mechanism of renal cancer stem cells (RCSCs) facilitates RCC treatment and prevention.

The Sonic Hedgehog (SHH) signaling pathway plays an essential role in self-renewal, tumor cell growth, drug resistance, metastasis, and recurrence of CSCs (Jeng et al., 2020). Stimulation of the SHH signaling pathway significantly boosted RCC cell proliferation and accelerated the EMT process, which promoted RCC progression (Behnsawy et al., 2013). Downregulation of the SHH pathway could inhibit the proliferation of RCSCs (Li et al., 2019).

Numerous studies have shown that CS is closely related to the occurrence and development of several cancers, such as bladder cancer (Jubber et al., 2023), gastrointestinal cancer (Yuan et al., 2023), and RCC (Znaor et al., 2015). CS could increase the risk of RCC by 50% in male and 20% in female smokers (Znaor *et al.*, 2015). We have explored the relationship between CS and RCC previously. We found that CS effectively promoted renal CSCs stemness by enhancing tumorphere formation and increasing the expression of renal CSCs markers (Qian et al., 2018). We also discovered that CS promoted RCC progression by promoting EMT (Zhang et al., 2020).

ΔNp63α is a significant regulator in maintaining the characteristics of CSCs. Studies have shown that ΔNp63α is required to regulate the SHH signaling pathway (Cao et al., 2008; Ge et al., 2023). Cao *et al*. (2008) found that overexpression of ΔNp63α in RCC could up-regulate the protein and mRNA levels of the SHH signaling pathway. Dietary diallyl trisulfide inhibited gastric cancer stemness by ΔNp63/SHH pathway (Ge *et al.,* 2023). Another study suggests that the IL-6/ΔNp63α/Notch axis plays a vital role in the long-term tobacco smoke exposure-induced acquisition of lung CSC-like properties (Xie et al., 2019). However, the role of ΔNp63α in CS-induced activity acquisition in CSCs remains largely unkown. Therefore, the present study aims to investigate the role of ΔNp63α/SHH signaling pathway in CS-triggered RCSCs and elucidate the underlying molecular mechanism, thereby providing valuable scientific insight for mechanism-oriented intervention research in RCSCs.

## Material and Methods

### Collection of patient samples

All samples were obtained from the Department of Urology, the Second Affiliated Hospital of Anhui Medical University, with the approval of the Ethics Committee of Anhui Medical University and the patient’s consent. During the collection, renal cancer tissues were cut off and stored in the refrigerator at -80 ℃ immediately, and the data were registered in the specimen bank. All patients had never received radiotherapy, chemotherapy, or targeted therapy. The pathological types were confirmed as clear cell RCC by two pathologists. Besides, smokers possess more than ten years of history. The study was conducted in the Second Affiliated Hospital of Anhui Medical University, Hefei, China, and was approved by the ethics committee of the hospital (No. LLSC20190660). All presentations of case reports have consent for publication.

### Cigarette smoke extract (CSE) preparation and cell culture

A filter-free 3R4F cigarette (9 mg tar and 0.76 mg nicotine/cigarette, Kentucky, USA) was burned to continuously instill CS at the rate of 5 min one cigarette through a glass syringe containing 10 ml of pre-warmed (37 ℃) serum-free medium. Then the CSE solution was adjusted to pH 7.4 and passed through a filter with a pore size of 0.22 µm. The solution was then referred to as 100% CSE solution.

Human clear cell renal carcinoma cell lines 786-O and ACHN were purchased from the Cell Bank of the Chinese Academy of Sciences (Shanghai, China). 786-O cells were cultured in RMPI-1640 medium supplemented with 10% fetal bovine serum and 1% penicillin-streptomycin, whereas ACHN cells were cultured in a cell incubator containing 5% CO_2_ at 37 °C and 5% CO_2_ at 37 °C using MEM medium.

### Tumor sphere formation assay

786-O, ACHN cells were seeded at 5000 cells/well into 24-well culture plates and cultured with SFM medium. The SFM medium was prepared using DMEM-F12, followed by adding 20 ng/ml EGF, 20 ng/ml bFGF, 5 µg/ml insulin, and 2% B27 for 5 consecutive days with semi-quantitative fluid exchanges every other day. Different concentrations of CSE were added to each well for 5 consecutive days, and only the number of tumor spheres with a diameter >50 μm was counted.

### Western blot experiments

The treated cells and tissues were lysed in RIPA buffer (Beyotime, Nanjing, China) containing 1% protease inhibitors and phosphatase. Protein concentration was determined by BCA Protein assay kit (Beyotime, Nanjing, China) followed by sodium dodecyl sulfate-polyacrylamide gel electrophoresis (SDS-PAGE) separation and transfer to PVDF membrane. Membranes were blocked with 5% skim milk powder at room temperature for 1 hour and incubated overnight at 4 °C with the following primary antibodies: ΔNp63α (13109S, Cell signaling technology, Massachusetts, USA), CD44 (15675-1-AP, Proteintech, Rosemont, IL, USA), Oct4 (60242-1-Ig, Proteintech, Rosemont, IL, USA), SOX2 (11064-1-AP, Proteintech, Rosemont, IL, USA), Gli2 (ab277800, Abcam, Cambridge, UK), Gli1 (ab217326, Abcam, Cambridge, UK), Smo (ab7817, Abcam, Cambridge, UK), Shh (ab53281, Abcam, Cambridge, UK), GAPDH (60004-1-Ig, Proteintech, Rosemont, IL, USA). Then followed by incubating with secondary antibodies. GAPDH was used as an internal control.

### Quantitative real-time polymerase chain reaction

TRIzol reagent was added to the treated cells for RNA extraction. The concentration was measured at a wavelength of 260 nm, and the cDNA was reverse transcribed according to the kit requirements (NovaBio, Massachusetts, USA) to obtain a concentration of no more than 50 mg/L cDNA. The liquids were then mixed according to the needs of the qPCR kit (NovaBio, Massachusetts, USA) and amplified using a two-step PCR reaction procedure. GAPDH was set as the internal control, and the relative expression of mRNA was calculated according to the 2^-(ΔΔCT)^ method.

### Detection of CD44 positive cells by flow cytometry

The treated cells were picked up and washed twice with precooled PBS solution. CD44 antibody was added, a secondary antibody was added after incubation at room temperature, and after incubation at room temperature in the dark, the cells were washed twice with PBS solution. Finally, the cells were resuspended in 200 µl PBS solution, and the number of CD44 positive cells was detected by computer operation.

### Cell viability assay

Cells were seeded into 96-well plates at 1000 cells/well density and semiquantitatively changed every other day in the SFM medium. Cell viability was measured using the Cell Counting Kit-8 (Sigma-Aldrich, Shanghai, China) according to the manufacturer’s instructions 5 days later.

### Immunohistochemistry

Paraffin tissue prepared in advance was sectioned on a microtome at a standard of 4.0 µm. Slides were then deparaffinized in xylene, hydrated in a graded ethanol series, and antigen repaired with citrate buffer. After 30 min block with serum at room temperature, primary antibody (ΔNp63α, 13109S, CST, 1:200; CD44, 15675-1-AP, Proteintech, 1:500) were incubated at 4 ℃ overnight and incubated at room temperature with secondary antibody for 30 min the next day. Cells were then briefly counterstained with diaminobenzidine (DAB), and nuclei were stained with hematoxylin. Finally, the slides were observed under a light microscope.

### Co-immunoprecipitation

The treated cells were collected, precooled lysate added, lysed sufficiently, and sonicated on ice for fragmentation. The cells were centrifuged at 14,000 × g for 10 min at 4 °C, and 5 µl Protein A and 5 µl Protein G were added to 500 µl of the supernatant. The cells were then centrifuged at 12,000 × g at 4 °C for 1 min, and 1 µg of antibody was added to the lysate. In contrast, IgG, a nonspecific immune homologous antibody, was used as a control and incubated overnight at 4 °C. An additional 5 µl Protein A and 5 µl Protein G were added, and the incubation was continued overnight at 4 °C. The samples were centrifuged at 12,000g for 1 min and washed with a cleaning solution, followed by centrifugation at 12,000 × g for 1 min and repeated cleaning three times. 20 µl of loading buffer was added, boiled at 100 °C for 5 min, and centrifuged at 14,000 × g for 1 min. The final samples were boiled for 5 min before electrophoresis and protein immunoblot analysis.

### Tumor formation experiment in nude mice

From the selection of Anhui Medical University Animal Experiment Center, 4 ~ 6 weeks of nude mice were obtained. The treated cells were mixed with 50 µl matrix gel and 50 µl PBS solution and then slowly injected subcutaneously into the armpit of mice at 1×10^6^ cells. The body weight of the mice and the eruption of tumors under the armpit were observed daily, and vernier calipers recorded the tumor size. The mice have sacrificed appropriately, and the tumors were removed and weighed.

### Statistical methods

Results presented in figures were representative from triplicate. Data were expressed as mean ± standard deviation after analyzing with SPSS 26.0 Software (SPSS, Inc., USA). One-way ANOVA was used for comparison of statistical differences among multiple groups, followed by the LSD significant difference test. In case of comparison between two groups, an unpaired Student’s *t*-test was used. Statistical significance was attributed at *p* < 0.05.

## Results

### Smoking promoted ΔNp63α expression in RCC patients and CSCs activity

**Figure 1-:**
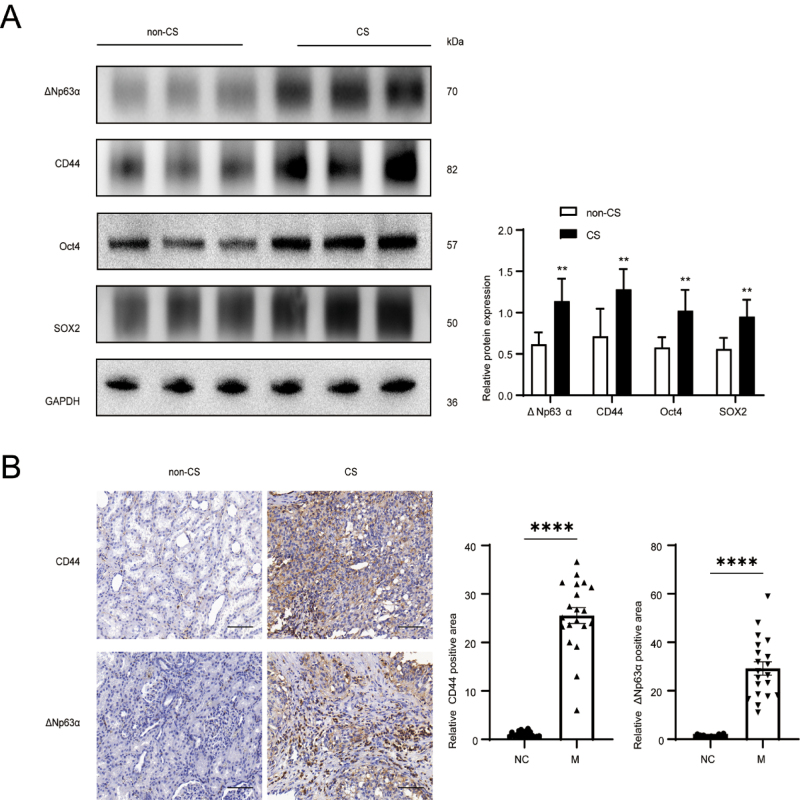
CS promotes the activity expression of ΔNp63α and CSCs markers in patients with kidney cancer. Western blot (A) and immunohistochemistry (B) detected the expression levels of ΔNp63α and RCSCs markers in the two groups (H&E, x200). *p<0.05, **p<0.01.

To investigate the correlation between ΔNp63α expression and smoking status in patients with RCC, 20 patients were enrolled in this study, including 10 smokers and 10 non-smokers (Table 1). All patients were diagnosed as clear cell RCC and received radical nephrectomy in our center. Western blot analysis showed that ΔNp63α expression was significantly higher in smokers than non-smokers. The expression levels of RCSCs markers, CD44, Oct4, and SOX2 in smoking patients were remarkably higher than their counterparts (Figure 1A , Figure S1), which were consistent with outcomes of immunohistochemistry (Figure 1B ).

**Table 1 -:** Clinical characteristics of patients (n=20).

Parameters	Number of cases
Gender	
Male	14（70%）
Female	6（30%）
Age	
≤50	5（25%）
>50~≤70	12（60%）
>70	3（15%）
Pack-years	
0	10（50%）
＞10-≤15	4（20%）
＞16	6（30%）
Tumor size (cm)	
≤4	7（35%）
>4~≤7	9（45%）
>7	4（20%）
Tumor stage	
pT1a	7（35%）
pT1b	8（40%）
pT2a	2（10%）
pT2b	1（5%）
pT3	2（10%）
TNM stage	
Ⅰ	15（75%）
Ⅱ	3（15%）
Ⅲ	2（10%）
Ⅳ	0（0%）

### RCSCs were enriched by suspension culture using serum-free medium (SFM)

**Figure 2 -:**
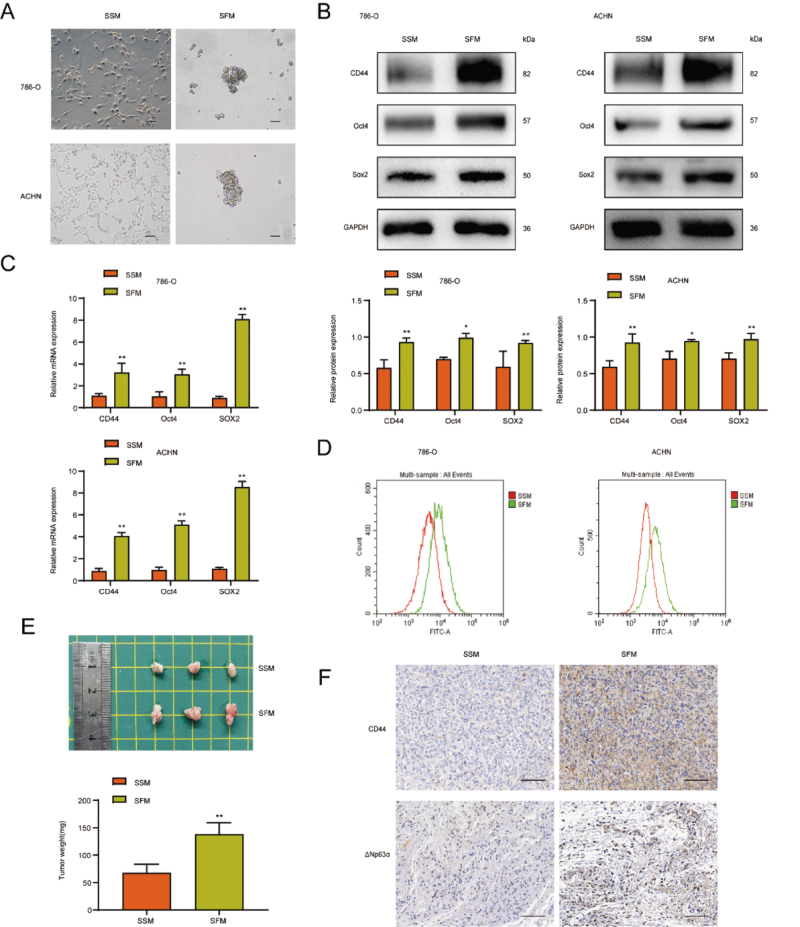
Enrichment of RCSCs by SFM. Renal cancer cells 786-O and ACHN were cultured with SFM and SFM for 5 days, respectively. (A) Microscopy was performed to observe the morphological changes of renal cancer cells (H&E, x100). The protein and mRNA expression levels of RCSCs markers were detected by western blot (B) and qRT-PCR (C). (D) The quantity of CD44-labeled positive cells in renal cancer cells was detected by flow cytometry. (E) Tumor size and weight were measured in SSM and SFM groups. (F) The expression of CD44 and ΔNp63α in SSM and SFM tissues was analyzed by immunohistochemistry (H&E, x200). *p<0.05, **p<0.01.

Renal cancer cells 786-O and ACHN were cultured in SFM and serum-supplied medium (SSM) for 5 days, respectively. We found that the morphology appearance of the cells in the SFM culture changed significantly from the original fusiform adherent growth to spherical suspension growth (Figure 2A ). Then we investigated the alterations at the cellular and molecular level by analyzing the protein expression levels of RCSCs markers CD44, Oct4, and SOX2. As expected, the expression levels were significantly elevated in the SFM treatment group (Figure 2B). Moreover, qRT-PCR analyses revealed similar changes in the mRNA levels of RCSCs markers in the SFM treatment group (Figure 2C ). In addition, flow cytometry of CD44-labeled renal cancer cells revealed a significant increase in CD44-positive cells (Figure 2D ), confirming the apparent enrichment of RCSCs after SFM treatment again. Renal cancer cells cultured in SSM and SFM were collected and injected subcutaneously into the axilla of nude mice. Compared with the SSM group, the SFM group significantly increased the tumor size and weight (Figure 2E ). Immunohistochemical results also showed that CD44 and ΔNp63α positive indicators in the SFM group were significantly increased, indicating that the activity of RCSCs was remarkably elevated (Figure 2F ). 

### CS promoted the activity of RCSCs

**Figure 3 -:**
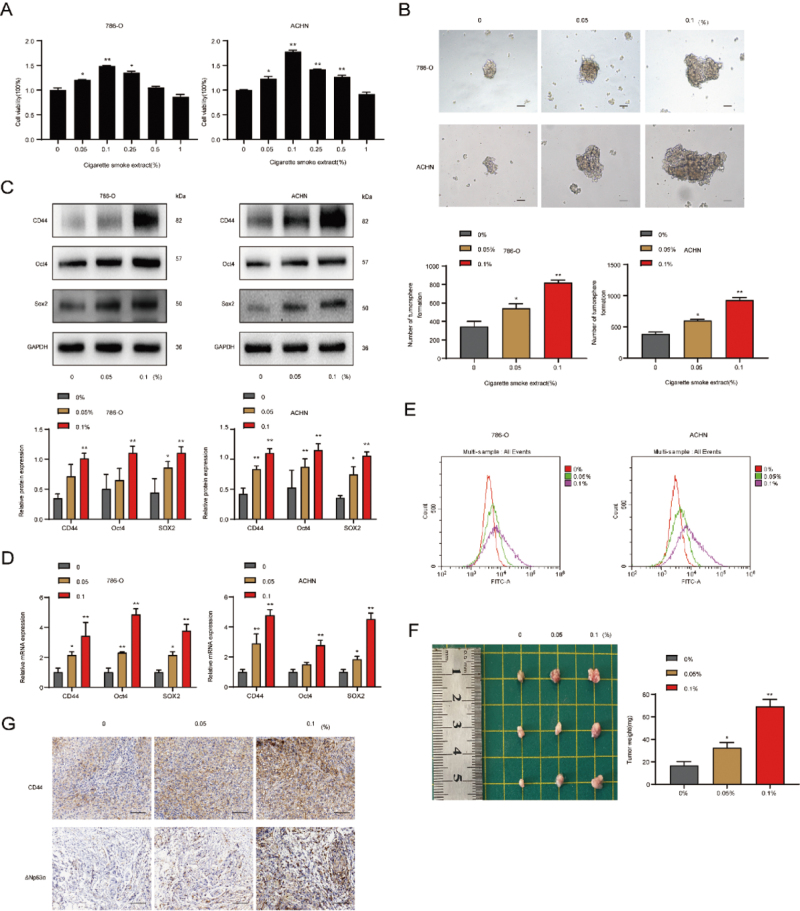
CS promotes the activity of RCSCs. RCSCs were administrated with various concentrations of CSE. (A) The effects of different concentrations of CSE on 786-O and ACHN tumor spheres were detected by CCK-8 assay. (B) The changes in the morphology and number of RCSCs treated with different concentrations of CSE were observed under a microscope (H&E, x100). The effects of CSE on the protein and mRNA expression levels of RCSCs markers were detected by western blot (C) and qRT-PCR (D). (E) Flow cytometry was utilized to determine the number of CD44-labeled positive cells in RCSCs treated with CSE. (F) The size and weight of tumors in the armpit of nude mice in the CSE-treated group were measured. (G) The immunohistochemical technique was used to detect the expression of CD44 and ΔNp63α in tumor tissues of the CSE-treated group (H&E, x200). *p<0.05, **p<0.01.

786-O and ACHN tumor spheres were treated with different concentrations of cigarette smoke extract (CSE) for 5 days, and cell viability was measured by CCK-8 assay. We found that cell viability enhanced significantly with increasing concentration, but cell viability began to decrease when the concentration exceeded 0.1% (Figure 3A ). Therefore, we selected CSE concentrations of 0, 0.05%, and 0.1% for subsequent experimental treatment. It was discovered that the diameter and number of 786-O and ACHN tumor spheres were positively correlated with the increase of CSE concentration (Figure 3B ). Next, we detected RCSCs treated with CS by western blot and qRT-PCR. Results showed that the protein (Figure 3C ) and mRNA (Figure 3D ) expression levels of RCSCs molecular markers were significantly elevated with the increase of CSE concentration. Moreover, a positive correlation between CSE concentration and the number of CD44-labeled positive cells was also detected by flow cytometry (Figure 3E ). Furthermore, similar results were demonstrated *in vivo* (Figure 3F-G ). Collectively, these results suggested that CS promoted the activity of RCSCs.

### ΔNp63α facilitated CS-induced activity of RCSCs

**Figure 4 -:**
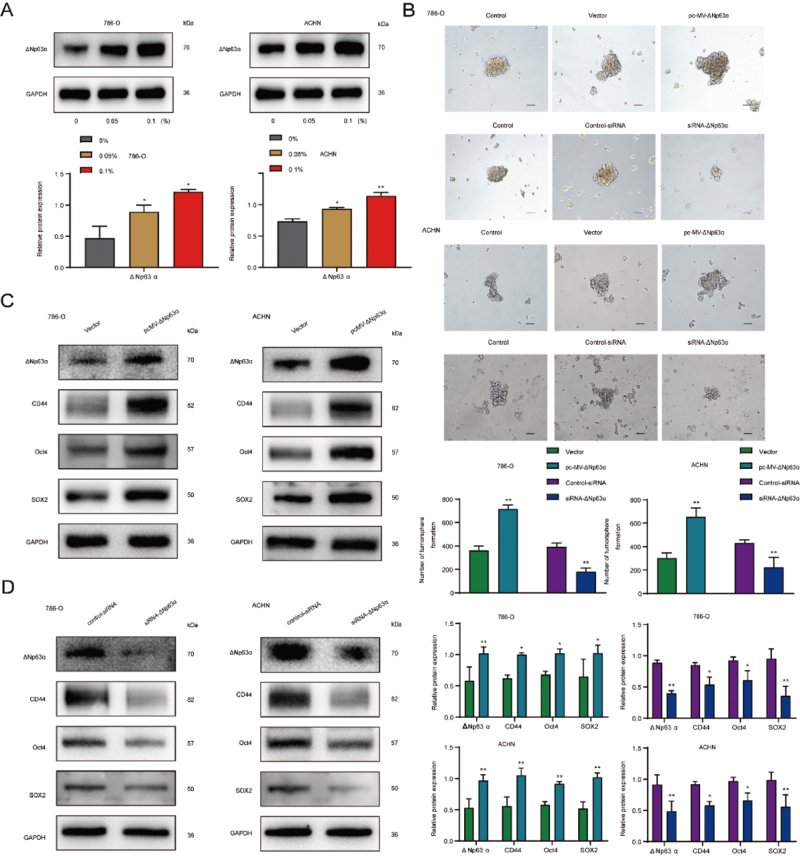
ΔNp63α promotes CSE-induced activity in RCSCs. 786-O and ACHN tumor spheres were transfected with overexpression plasmids and targeted small interfering RNA. (A) Western blot analysis of ΔNp63α expression in CSE-treated tumorspheres. (B) Tumor spheres transfected with overexpression plasmid and siRNA were observed by microscopy (H&E, x200). (C) western blot analysis of the expression of ΔNp63α, CD44, Oct4, and SOX2 in tumorspheres transfected with overexpression plasmids. (D) Western blot analysis of the expression of ΔNp63α, CD44, Oct4, and SOX2 in the tumorspheres after siRNA transfection. *p<0.05, **p<0.01.

Our results above have shown that ΔNp63α expression is closely associated with smoking status in renal cancer patients and ΔNp63α expression is high in 786-O and ACHN tumor spheres with CSE treatment (Figure 4A ). To further investigate whether ΔNp63α plays a role in CS-induced RCSCs, the ΔNp63α plasmid was transfected into the CS-induced 786-O and ACHN tumor spheres, and the ΔNp63α overexpression model was established by culturing in an SFM medium for 4 days. Microscopy was performed to determine the cellular morphology alterations. We found that 786-O and ACHN tumor spheres were bigger in diameter and more numerous after plasmid transfection than those in the control group (Figure 4B ). Moreover, ΔNp63α overexpression of 786-O and ACHN tumor spheres significantly increased the protein expression levels of RCSCs markers CD44, Oct4, and SOX2 compared with the control group (Figure 4C ). In contrast, the knockdown of ΔNp63α in tumor spheres with siRNA-ΔNp63α resulted in a smaller tumor diameter and a reduced number of tumor spheres (Figure 4B ). It suppressed the protein expression levels of RCSCs markers CD44, Oct4, and SOX2 (Figure 4D ). These observations demonstrated a critical role of ΔNp63α in regulating the activity of RCSCs induced by CS.

### SHH signaling was stimulated in the CS-induced acquisition of activity in RCSCs

**Figure 5-:**
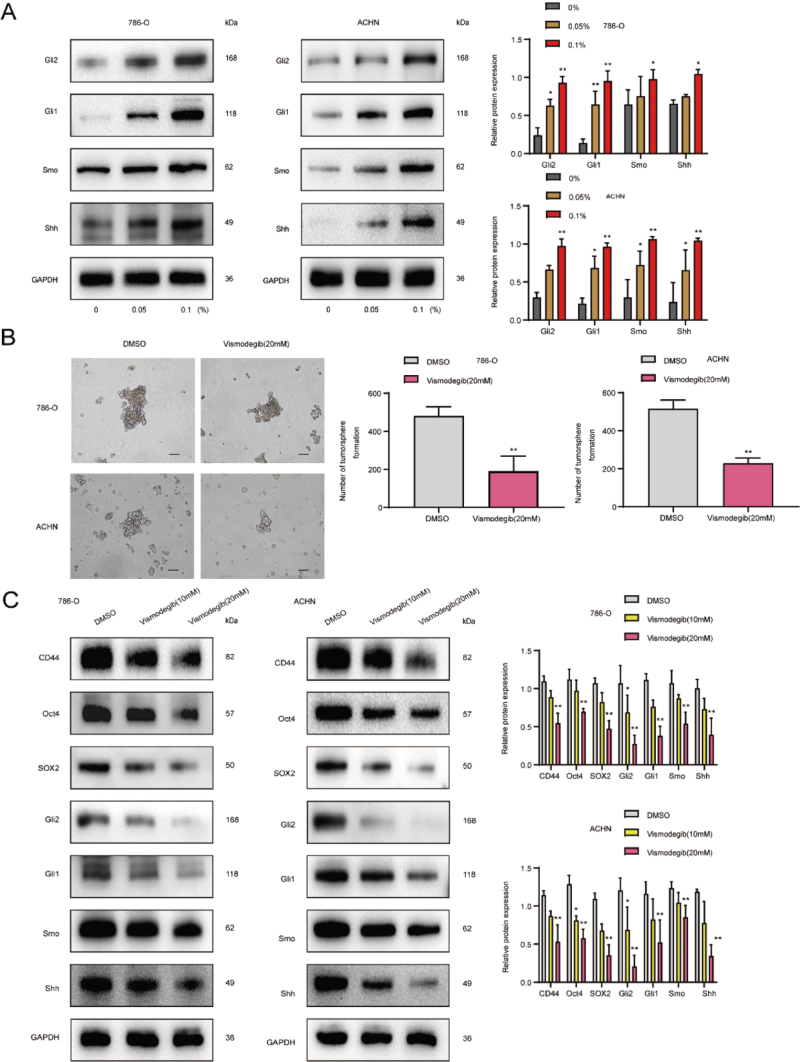
SHH signaling pathway is promoted in the CSE-induced acquisition of activity in RCSCs. (A) Western blot analysis of the expression levels of SHH signaling pathway-related proteins Shh, Smo, Gli1, and Gli2 in CSE-treated RCSCs. (B) Changes in the size and number of tumor spheres after treatment with SHH signaling pathway inhibitor Vismodegib were observed under a microscope (H&E, x100). (C) The expression levels of CD44, Oct4, and SOX2 in tumor spheres treated with Vismodegib were detected by Western blot. * ***p*** <0.05, ** ***p*** <0.01.

Western blot showed that the protein expression levels of Shh, Smo, Gli1, and Gli2 in 786-O and ACHN tumor spheres were increased after treatment with different concentrations of CSE (Figure 5A), indicating the stimulation of the SHH signaling pathway. Then we explored the role of SHH signaling in CSE-induced RCSCs by using Vismodegib (Erivedge, Genentech, USA), an SHH signaling inhibitor, and found that Vismodegib treatment significantly reduced the size and number of RCSCs spheroids (Figure 5B ). Next, we detected that Vismodegib reduced protein expression of CD44, Oct4, and SOX2 in RCSCs 786-O and ACHN remarkably (Figure 5C ). These results suggested that SHH signaling played a vital role in CSE-induced RCSCs.

### ΔNp63α positively regulated CS-induced activity of RCSCs via the SHH pathway

**Figure 6 -:**
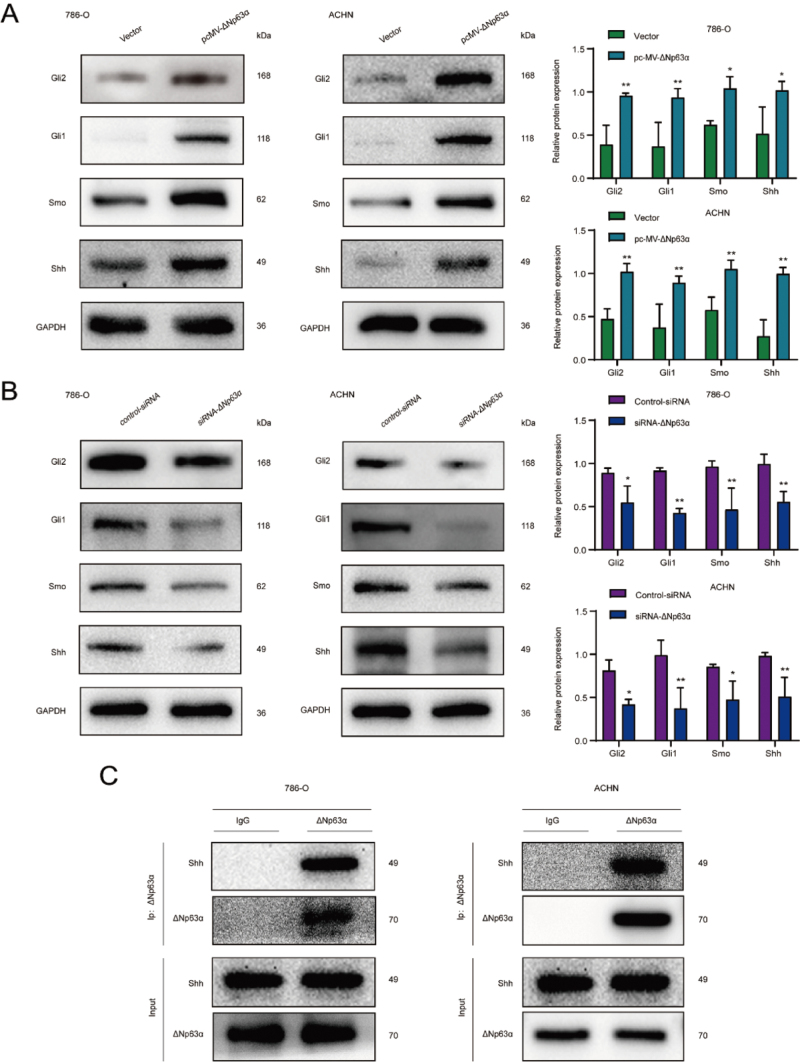
ΔNp63α positively regulates CS-induced activity in RCSCs through the SHH pathway. (A, B) Western blot was used **to determine** the expression levels of SHH signaling pathway-related proteins Shh, Smo, Gli1, and Gli2 in tumor spheres with or without ΔNp63α overexpression. (C) Co-immunoprecipitation assay examined the interaction between ΔNp63α and the SHH signaling pathway. * ***p*** <0.05, ** ***p*** <0.01.

**Figure 7 -:**
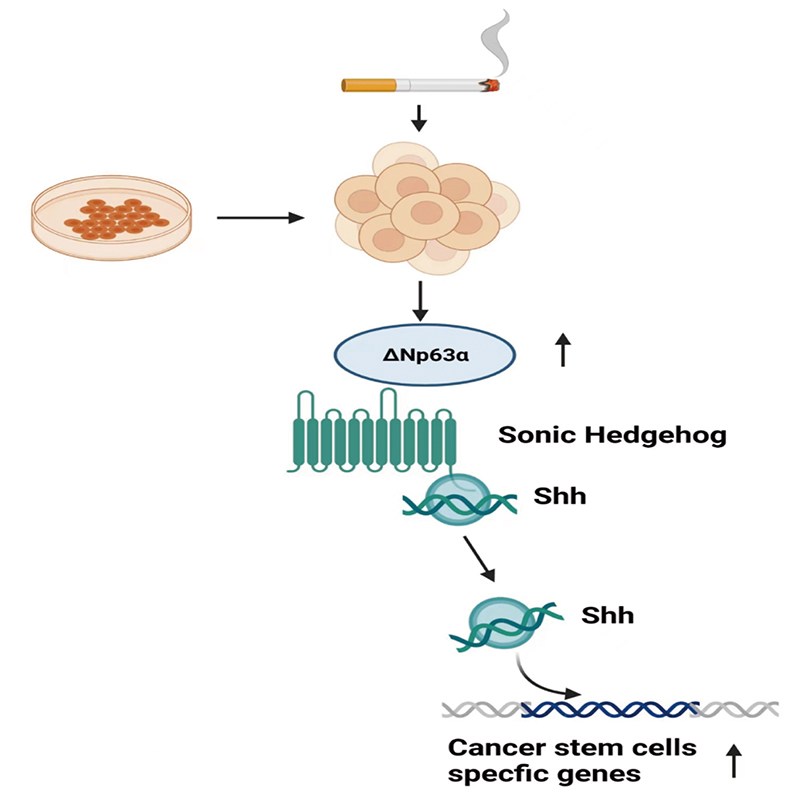
Schematic diagram.

To explore whether ΔNp63α regulates CS-induced activity of RCSCs through the SHH pathway, we transfected ΔNp63α overexpression plasmid and siRNA into CS-induced 786-O and ACHN tumorspheres. Western blot detected the expression levels of SHH signaling pathway-related proteins Shh, Smo, Gli1, and Gli2 in the transfected cells. As expected, the expression level of SHH signaling pathway-related proteins was increased in the overexpressed ΔNp63α cells (Figure 6A ). In contrast, inhibition of ΔNp63α reduced the expression of the SHH signaling pathway (Figure 6B). Subsequently, to verify this relationship, we performed a co-immunoprecipitation assay and verified the interaction of ΔNp63α with SHH signaling at the transcription level (Figure 6C). Figure 7 presents the mechanism involved in the CS-mediated induction through a schematic diagram. Our results revealed that ΔNp63α modulated CS-induced activity in RCSCs via the SHH pathway.

## Discussion

In recent years, the role of CSCs in the development and progression of RCC has been demonstrated (Lasorsa et al., 2023). Ma et al. (2023) indicated that inhibition of the expression of a significant marker of CSCs, i.e., CD44, may be a new therapy to suppress ccRCC progression. Oct4, a stem cell transcription factor, and its overexpression was closely related to the progression of several malignancies, including ovarian cancer (Xie et al., 2022), prostate cancer (Yasumizu et al., 2020), and renal cancer (Zhang et al., 2023). Overexpression of SOX2 is associated with cancer development, poor prognosis, and chemoresistance in several types of cancers (Mirzaei et al., 2022). CS is an improtant risk factor of RCC. ΔNp63α has been demonstrated to be a vital RCC-associated gene. However, the molecular mechanism of ΔNp63α in CS-induced acquisition of activity in RCSCs remains unclear. We found that ΔNp63α expression in the RCC tissues of smokers was higher than that in the RCC tissues of non-smokers, and the levels of CSCs markers were significantly elevated in the RCC tissues of smokers.

In the presence of growth factors, culturing adherent cells in low-attachment plates with the serum-free medium can improve tumorigenicity and enrich cell cultures with stem cell characteristics (House et al., 2015). In this study, we cultured 786-O and ACHN renal cancer cells with SFM to increase spherical cells with stem cell characteristics, detected the expression levels of CSCs markers, and found that the protein expression levels of CSCs markers CD44, Oct4, and SOX2 increased. Flow cytometry showed that the number of CD44 labeled positive cells grew. Therefore, it is concluded that RCSCs are enriched after SFM treatment.

There is increasing evidence that CS can increase the activity of CSCs. CS contains various chemical components, among which nicotine is closely related to carcinogenesis (Li et al., 2023). *In vitro*, we found that CS at a specific concentration could increase the diameter and number of 786-O and ACHN tumor spheres, and the expression level of RCSCs markers (CD44, Oct4, SOX2) was significantly increased. *In vivo*, the tumor size and weight of nude mice increased after CSE treatment, and the expression of molecular markers of RCSCs was elevated. Our results suggested that CS promotes the stem cell activity of RCSCs.

ΔNp63α overexpression plays a vital role in maintaining the activity of CSCs in several cancers (Cao et al., 2008; Ge et al., 2023). Cao *et al*. (2023) indicated that low-dose human phthalate exposure promoted the stem cell properties of breast CSCs in an ΔNp63α and SHH-dependent manner. The mechanism between CS and ΔNp63α has been poorly studied. In CS-exposed lung cancer cells, ΔNp63α regulated COX-2 expression and promoted inflammatory and tumorigenesis processes (Ratovitski, 2010). ΔNp63α/IRF6 interplay regulates nitric oxide synthase-2 transcription in head and neck squamous cell carcinoma cells and immortalized oral keratinocytes, which reveals its important role in the autophagy-related cancer cell response (Ratovitski, 2011). ΔNp63α promoted the acquisition of lung CSC-like properties in long-term tobacco smoke exposure-transformed Human bronchial epithelial cells (Xie et al., 2019). However, the role of ΔNp63α in CS-induced RCSCs remains unknown. Our results showed increased ΔNp63α expression levels in RCC tissues from smokers. ΔNp63α overexpression enlarged tumor spheres in diameter and number and expressed higher levels of RCSCs markers. Moreover, ΔNp63α knockdown reduced tumor spheres in diameter and number and reduced the levels of RCSCs markers. These data revealed that ΔNp63α was essential for regulating stem cell activity in CS-induced RCSCs. 

The SHH pathway plays a crucial role not only in embryonic organ development but also in the maintenance of CSC activity, tumorigenesis, metastasis, recurrence, and treatment (Jeng et al., 2020). In breast cancer, ΔNp63α regulated the expression of Shh, SMO, Gli1, and Gli2 genes by directly binding to their gene regulatory regions and ultimately promoting the activation of signaling, whereas downregulation of ΔNp63α diminished SHH signaling molecules (Cao et al., 2023). Similar findings were detected in RCC cells (Cao *et al.*, 2008). In this study, we observed that the protein expression levels of Shh, Smo, Gli1, and Gli2 were increased in 786-O and ACHN tumor spheres under CS treatment, and suppressed the ability of tumor sphere formation. The expression levels of CSCs markers were decreased after the administration of SHH signaling pathway inhibitor Vismodegib. Further studies found that ΔNp63α overexpression increased the expression of Shh, Smo, Gli1, and Gli2. Subsequent Co-IP experiments verified the interaction of ΔNp63α with the SHH signaling pathway. These observations demonstrated that ΔNp63α activated SHH signaling and promoted CS-induced activity in RCSCs.

The present study has several limitations. First, the small numbers of patients in the study might have led to possible selection bias. Second, other signaling pathways, Notch pathway for example, have also been found to be closely related to ΔNp63α and contribute to cancer progression, which warrants further study (Xie et al., 2019).

## Conclusion

In conclusion, the present study demonstrated that ΔNp63α positively regulates the CS-induced activity of RCSCs via the SHH pathway, which may provide novel ideas and options for treatment of CS-related RCC.

## Data Availability

 The data that support the findings of this study are available from the corresponding authors (zhangtao@ahmu.edu.cn or m15077916345_1@163.com) upon reasonable request.

## Supplementary material

The following online material is available for this article:

Figure S1 - Western blot analysis of the expression levels of RCSCs markers, CD44, Oct4, and SOX2 in clinical patients.
