# Supplementary material for: ΔNp63α promotes cigarette smoke-induced renal cancer stem cell activity via the Sonic Hedgehog pathway
Source: Genet Mol Biol. 2024 Jul 1;47(2):e20230347. doi: 10.1590/1678-4685-GMB-2023-0347 (PMC11234498; doi:10.1590/1678-4685-GMB-2023-0347)
Supplement: Figure S1 - [file 1415-4757-GMB-47-02-e20230347-s1.pdf]

Genetics and Molecular Biology

https://doi.org/10.1590/1678-4685-GMB-2023-0347

Supplementary Material to "ΔNp63α promotes cigarette smoke-induced renal cancer stem cell activity via the Sonic Hedgehog pathway"

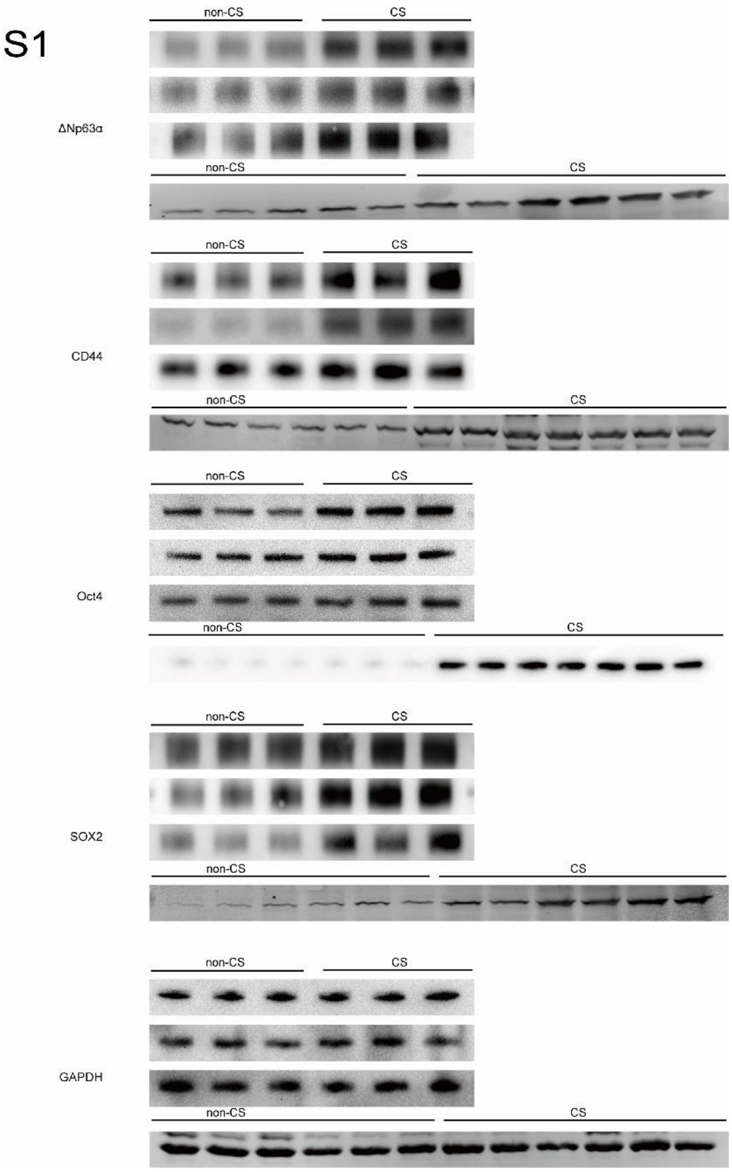

Figure S1 - Western blot analysis of the expression levels of RCSCs markers, CD44, Oct4, and SOX2 in clinical patients.
